# Beta-band power is an index of multisensory weighting during self-motion perception

**DOI:** 10.1016/j.ynirp.2022.100102

**Published:** 2022-05-28

**Authors:** Ben Townsend, Joey K. Legere, Martin v. Mohrenschildt, Judith M. Shedden

**Affiliations:** a Psychology, Neuroscience & Behaviour, Science, McMaster University, Hamilton, Ontario, Canada; b Computing and Software, Engineering, McMaster University, Hamilton, Ontario, Canada

**Keywords:** Self-motion perception, Vestibular, Motor cortex, Event-related spectral power, Alpha power, Beta power, Theta power

## Abstract

Human self-motion perception largely relies on the integration of the visual, vestibular and proprioceptive systems. Much behavioral research has been conducted in order to understand this integration process; however, little is known about the online processes in humans during self-motion perception. Of the few studies to physically move human participants with full-body motion while recording the brain, most have used EEG due to its relative mobility. Past research provides evidence that multisensory self-motion perception elicits theta, alpha, and beta oscillations. It is important, however, to understand the individual contribution of each sense to fully understand how these oscillatory frequencies contribute to self-motion perception. To our knowledge, there has yet to be a study that directly compares the EEG correlates of visual self-motion with a no-motion physical input, versus physical-self motion with a no-motion visual input. We recorded event-related spectral power within a motion simulator controlled by a MOOG Stewart platform. Participants were given a visual or physical stimulus and made heading direction judgments. Compared to physical-only trials, visual-only trials produced earlier theta ERS and alpha ERD early in the trial, and more robust beta ERS late in the trial. We suggest beta-band power is likely associated with the process of visual-vestibular weighting. Moreover, within the right motor area, we found differences in theta power associated with left versus right headings. Theta ERS in the right motor area appears to be associated with heading processing for both the visual and vestibular systems but is minimally affected by multisensory weighting.

## Highlights

• Visual and physical self-motion cues elicit ERSP within the same spectral bands.
• Theta ERS in the right motor area is sensitive to heading in both modalities.
• The beta band is associated with the process of visual-vestibular weighting.

## 1 Introduction

The perception of self-motion has been of interest to scientists from a broad range of disciplines for the past several decades. These varying areas of study include (but are not limited to) spatial navigation (Moffat, 2009), fall prevention (Lupo and Barnett-Cowan, 2018; St. George and Fitzpatrick, 2011) and driver and pilot training (De Winter, Dodou & Mulder, 2012). It has been well established that self-motion perception is a multisensory phenomenon. The senses involved in this phenomenon include the visual, vestibular, proprioceptive, tactile and auditory systems (Gu et al., 2008). The vestibular system detects linear and rotational movements of the head (for review see, Angelaki and Cullen, 2008), while the proprioceptive system detects body movements through the displacement of receptors in muscles (for review see, Proske and Gandevia, 2012). Together the vestibular and proprioceptive systems allow humans and other organisms to experience physical self-motion, or inertial motion, which then integrates with the available self-motion information from the visual and/or auditory systems (Gu et al., 2008).

Researchers in the aviation and driver training industries have paid particularly close attention to the integration processes of visual and physical self-motion. This research emphasis is likely due to Transport Canada (TC) and the Federal Aviation Administration's (FAA) requirements for the highest fidelity physical-motion systems for military pilot training, despite decades of mixed results regarding their training effectiveness (Bürki-Cohen and Go, 2005). Moreover, high-fidelity motion-based platforms for pilot training are extremely expensive and labor intensive to maintain. This lack of conclusive evidence in favor of physical motion for pilot training may be, in part, because the benefit of physical motion during training is limited to specific flight tasks. For example, compared to visual-motion cues only, some studies demonstrated a training benefit of incorporating physical-motion cues that simulated disturbance motion (external forces such as wind gusts, engine failure, etc.), but not for physical-motion cues that simulated correlated motion (movements of the vehicle controlled by the operator; (De Winter, Dodou & Mulder, 2012; O’Malley, Rajagobal, Grundy, v. Mohrenshildt & Shedden, 2016). High-fidelity motion systems continue to be required in TC and FAA policy primarily due to expert pilots' subjective preference for high-fidelity motion systems (Jones, 2016; Miletović et al., 2017). Given the perceived benefits and the high cost of motion simulators, it is important to improve understanding of the specificity of the contribution of physical-motion cues to effective training.

There exists substantial behavioral research focusing on how humans perceive (Harris and Barnes, 1987), integrate (Butler et al., 2015) and learn (Hays et al., 1992) from cues to self-motion in simulated environments. Neuroimaging research exploring the online processes related to perceiving and interpreting cues to physical self-motion is sparser. This lack of research is likely due to the technological difficulties of recording from the brain while participants are physically moving. Neuroimaging techniques like functional magnetic resonance imaging (fMRI), positron emission topography (PET), and electroencephalography (EEG) generally require participants to stay as stationary as possible (Lopez et al., 2012). Of these methods, EEG provides the most promise for reliable brain measures during physical movement because the equipment can move with the participant (unlike fMRI or PET).

Early studies exploring the online processes of self-motion perception primarily used rotatory chairs to present motion stimuli, while recording EEG. This method produces motion on the yaw axis, which stimulates the horizontal semicircular canals. Cortical activity was typically analyzed in the time domain, which demonstrated a number of perturbation-evoked potentials (PEPs; Probst et al., 1997; Schneider et al., 1996). A relatively recent review of PEPs (Varghese et al., 2017) concluded that PEPs are distributed over fronto-centro-parietal areas. According to Varghese et al. (2017), the time course of a PEP is composed of a small positive potential (P_1_) that peaks around 30–90 ms after perturbation onset, this is followed by large negative potential (N_1_) peaking around 90–160 ms, and finally, positive (P_2_) and negative (N_2_) potentials between 200 and 400 ms. A study by Varghese et al. (2014) examined cortical responses to vestibular perturbations in the frequency domain. They determined that the PEP N_1_ response is composed of activity in the delta (1–4 Hz), theta (4–7 Hz), alpha (8–12 Hz), and beta (13–30 Hz) bands.

Townsend, Leger, O’Malley, v. Mohrenschildt and Shedden (2019) used linear translation cues to examine event-related spectral power (ERSP) signatures of multisensory visual and vestibular/proprioceptive (physical) self-motion perception. They observed theta, alpha, and beta oscillations in response to simultaneous visual- and physical-motion stimuli. Participants attended to either the visual-motion and ignored physical-motion cues, or attended to the physical-motion and ignored visual-motion cues. The task was to make heading judgements (left or right) based on the direction of perceived self-motion indicated by the stimulus in the attended modality. This design allowed an examination of ERSP differences elicited by attention allocation. Attending to the visual-motion stimulus (while ignoring the physical-motion stimulus) evoked earlier theta event-related synchronization (ERS) and alpha event-related desynchronization (ERD), whereas attention to the physical-motion stimulus (while ignoring the visual-motion stimulus) evoked longer-lasting and more powerful beta ERD, all in the motor area. Townsend et al. (2019) were also able to demonstrate a congruency effect in the theta band. Incongruent motion stimuli elicited significantly stronger theta ERD power in the occipital area. Past research supports the idea that these three oscillatory frequencies are commonly recorded in the motor area during sensorimotor processing and output. Functionally, theta ERS is associated with heading processing (Burgess, 2008), alpha ERD is commonly linked to selective attention (Klimesch, 2012), and beta ERD reflects processes involved in preparing and executing motor output (Gaetz et al., 2010; Ofori et al., 2015; Tzagarakis et al., 2010).

Importantly, the Townsend et al. (2019) study showed individual differences in visual-vestibular weighting. Some participants found it more difficult to attend to the physical-motion cues and ignore the visual-motion cues, evidenced by poor accuracy identifying physical-motion heading direction on the incongruent trials. Participants who performed with high accuracy in that condition exhibited greater beta ERD power than those with poor accuracy. We believe that these differences in accuracy may be due to individual differences in visual-vestibular weighting, with low-accuracy participants having greater bias towards visual information. The beta band observations may point to a network that plays a key role in the weighting process. To further clarify the relationship between beta ERD power and a multisensory weighting process, it would be helpful to compare the Townsend et al. (2019) results with a design that does not present visual and physical cues simultaneously. Thus, the present study examined visual-only and physical-only conditions to compare the neural signatures revealed by theta, alpha and beta oscillations for each sensory system. One limitation of Townsend et al. (2019) is that there was no way of isolating potential reflexive cortical activity from any of the conditions because there was physical motion in all conditions. By isolating the visual from vestibular modalities and then directly comparing the induced cortical activity, the present experimental design allowed us to distinguish oscillatory frequencies caused by potentially reflexive movements (cortical activity that occurs only in the physical-only condition) versus those that index more general processes related to self-motion perception (cortical activity that occurs in both conditions) such as direction processing, attention, and multisensory weighting.

The goals of the present study were threefold. First, we wanted to determine whether the previously discussed oscillatory frequencies were affected by multisensory weighting. In the Townsend et al. (2019) experiment, two strong self-motion stimuli were presented simultaneously, therefore it was not possible to determine whether the observed power changes were due to weighting, or other factors such as unisensory processing, or attention-related functions. The present study used the same task and stimuli as Townsend et al. (2019) but differed in how the stimuli were (theoretically) perceptually weighted. We introduced far more weighting bias towards each stimulus in the present study, because only one stimulus was presented at a time. Based on our previous findings, we predicted that the beta band may be most affected by multisensory weighting. We hypothesized that if the beta band is in fact associated with multisensory weighting, we would find much different beta power changes between conditions (more so than theta and alpha) in the present study versus Townsend et al. (2019) due to the different weighting demands between studies.

Second, we used the heading discrimination task to explore whether any frequencies were sensitive to right versus left heading direction. We hypothesized that theta ERS was most likely to be direction sensitive, based on the sensitivity of theta to spatial incongruency in the Townsend et al. (2019) study. This congruency effect is only possible if theta oscillations are sensitive to heading in both the visual and vestibular modalities.

Third, most of the previous studies that recorded cortical activity during physical motion (e.g., Ditz et al., 2020; Townsend et al., 2019; Varghese et al., 2014) either provided simulated visual cues to motion on a display screen, or provided egocentric visual cues as participants moved through an environment with stable objects. These experimental designs provided simultaneous physical and visual cues to motion, and therefore did not isolate cortical activity based on sensory input. In the present study, participants were fully enclosed inside the cabin, thus all of the objects within the visual field moved with them during physical motion, minimizing any visual cues to motion in the physical-only condition. This design allowed us to isolate cortical responses to physical versus visual motion. This element of the design is particularly important for gaining insights into cortical activity induced by compensatory reflexes in response to physical motion. If our physical-motion stimuli are inducing strong reflexive responses, there will be powerful, and consistent differences in the motor cortices, between the physical-only and visual-only conditions directly following stimulus onset (Peterson and Ferris, 2018).

## 2 Materials and methods

### 2.1 Participants

Eleven participants (8 female) were recruited from the McMaster University psychology participant pool and the McMaster community. Ages ranged from 18 to 28 years (*M* = 19, *SD* = 3.07). Those recruited from the participant pool were compensated with course credits. All participants reported normal or corrected-to-normal vision, and no known problems with vertigo, motion sickness or claustrophobia. This experiment was approved by the Hamilton Integrated Research Ethics Board and complied with the Canadian tri-council policy on ethics.

### 2.2 Stimuli

#### 2.2.1 Visual-motion stimuli

Visual stimuli were presented on a 43-inch LCD panel at a resolution of 1920 × 1080 (1080p) and refresh rate of 60 Hz; with 51 inches between the screen and the participant. The screen subtended a horizontal visual angle of 41°. The visual-motion stimuli were presented in the same cabin as the physical-motion stimuli.

**Fig. 1:**
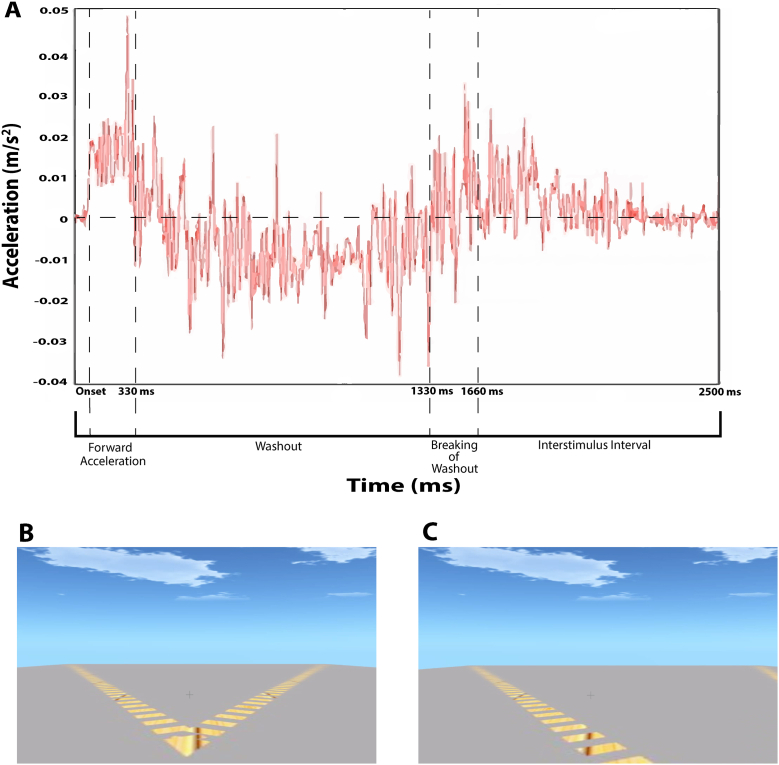
Time course of physical- and visual-motion stimuli. Panel A shows an example of the profile of physical motion measured during a single trial by an accelerometer (red line); the variance shown is due to the high sensitivity of the accelerometer. The x-axis represents time and the y-axis represents acceleration (g = m/s^2^). The acceleration profile is similar for 35° left and 35° right physical-motion trials. Panel B shows the visual display before the onset of visual motion; at this point the participant does not know whether visual motion will indicate travel along the left or right track. Panel C shows a still picture of the dynamic visual-motion display at approximately 1s after visual onset of a left visual motion trial. (For interpretation of the references to colour in this figure legend, the reader is referred to the Web version of this article.)

The visual display consisted of a fixation cross in the centre of the display and two tracks on a grey surface along which the perception of self-motion would occur. Each track consisted of a series of yellow dashes perpendicular to the length of the track, drawn in perspective to a vanishing point so that the track appeared to extend into the distance. Each track demarked a trajectory beginning at the lower center of the display; one track veered left by 35° and one track veered right by 35°. Both tracks together subtended a horizontal visual angle of 33.69°. The scene was demarcated by the grey surface below (upon which the tracks laid) the horizon and a blue sky with white clouds above, accentuating the perception of traveling along a track into the distance. The perception of self-motion was achieved via a first-person viewpoint animation that simulated moving forward along one of the tracks (two temporal snapshots are illustrated in Panels B and C of Fig. 1). The timing of each visual-motion trial was a forward motion along the left or right track for 700 ms followed by a pause at the end of the track for 1200 ms. The visual display was then reset to the starting position, ready for the next trial.

In the physical-motion task, participants saw the fixation cross, grey surface, and blue sky/clouds only; the yellow tracks were not present and there were no visual cues to self-motion.

#### 2.2.2 Physical-motion stimuli

A motion simulator provided physical motion stimuli. An enclosed cabin equipped to provide an immersive virtual environment was supported by a MOOG © Stewart platform with capability of six-degrees-of-freedom motion (Moog series 6DOF2000E; see Inline Supplementary Figure A1). Participants were seated in a bucket-style car seat fixed to the floor of the cabin.

Each physical-motion stimulus consisted of a linear translation of the cabin, moving forward at an angle of 35° left or 35° right. The duration of the movement was 330 ms at 0.1 g (the longest our motion simulator could be moved given the spatial restrictions of the motion platform). This surge was followed by a corresponding washout for 1330 ms which returned the cabin to the original position (see Panel A in Fig. 1). The acceleration intensity was selected based on preliminary testing to achieve a clear perception of forward motion within the spatial restrictions of the movement of the platform while minimizing compensating movements of the head, neck or upper body (Townsend et al., 2019). Physical forward accelerations were well above vestibular thresholds of 0.009 g, as discussed by Kingma (2005). The motion force, s(t), was described by:

$$s\left(t\right)=\left\{ \begin{array}{l} A_{1}\;0\;\leq t\;\leq t_{p}\\ -A_{2}\;t_{p}\;\leq t_{b}\\ A_{2}\;t_{b}\;\leq t\;\leq t_{e}\\ 0\;else \end{array}\right.$$

where t represents time in seconds, tp represents present time, tb represents the breakpoint and te represents the end time. A1 describes the initial forward acceleration, -A2 describes the initial (backwards) acceleration of the washout, and A2 describes the deceleration of the washout. Acceleration was measured using an Endevco accelerometer (model number 752A13), calibrated to approximately 1 mV/g sensitivity.

### 2.3 Procedure

The entire session was between 1.5 and 2 h in duration. The timeline of the session included collection of demographic information (age, gender, and handedness), followed by completion of 30 practice trials (2 min), application of EEG electrodes (25 min), completion of two experimental blocks (40–50 min), and participant cleanup and debriefing (15 min).

Visual-motion trials and physical-motion trials were blocked (199 trials per block); block order was counterbalanced. Participants were provided with earplugs and white audio noise was played inside the simulator to mask the sound of the motors during the physical-motion trials. Although the motion simulator was parked during the visual-motion trials (i.e., the cabin remained stationary), earplugs and white noise were applied in both visual- and physical-motion conditions for consistency. The interior of the cabin was monitored via a video camera.

Participants held a button box in their lap so that left and right thumbs rested on two response buttons. Their task was to respond with a button press to indicate the direction of perceived self-motion (left or right). Participants maintained fixation on the fixation cross throughout each trial and were provided with a “blink” break every 15 trials.

### 2.4 EEG data acquisition

EEG data were collected using the BioSemi ActiveTwo electrophysiological system (www.biosemi.com) with 128 sintered Ag/AgCl scalp electrodes. An additional four electrodes recorded eye movements (two placed laterally from the outer canthi and two below the eyes on the upper cheeks). Continuous signals were recorded using an open pass band from direct current to 150 Hz and digitized at 1024 Hz.

### 2.5 EEG preprocessing

All processing was performed in Matlab 2014a using functions from EEGLAB (Delorme and Makeig, 2004) on the Shared Hierarchical Academic Research Computing Network (SHARCNET: www.sharcnet.ca). A flowchart illustrating the signal-processing pipeline can be found in the supplementary materials (see Inline Supplementary Figure A2). EEG data were band-pass filtered between 1 and 50 Hz, and epoched from 1000 ms pre-stimulus to 2000 ms post-stimulus. Each epoch was baseline corrected using the whole-epoch mean (Groppe et al., 2009). After referencing, channels with a standard deviation exceeding 200 μV were interpolated (overall, only one channel was interpolated). Bad epochs were rejected if they had voltage spikes exceeding 500 μV, or were rejected by EEGLAB's joint probability functions (Delorme et al., 2007).

Single-subject EEG data were submitted to an extended Adaptive mixture independent component analysis (AMICA) with an N – (1 + interpolated channels) Principal Components Analysis reduction (Makeig et al., 1996). Decomposing an EEG signal into independent components (ICs) allows for analysis of each individual signal produced by the brain that would otherwise be indistinguishable. Following AMICA, dipoles were fit to each IC using the fieldtrip plugin for EEGLAB (Oostenveld et al., 2011). ICs for which the dipole fit explained less than 85% of the weight variance, or whose dipoles were located outside the brain, were excluded from further analysis. On average, 22.64 ICs per subject were excluded from analysis.

### 2.6 ERSP measure projection analysis

Event-related spectral power (ERSP) was computed for each of the remaining ICs. Fifty log-spaced frequencies between 3 and 50 Hz were computed, with 3 cycles per wavelet at the lowest frequency up to 25 at the highest. Measure projection analysis (MPA) was used to cluster ICs across participants using the Measure Projection Toolbox for MATLAB (Bigdely-Shamlo et al., 2013). MPA is a method of categorizing the location and consistency of EEG measures, such as ERSP, across single-subject data into 3D domains. These domains are subsets of ICs that are identified as having spatially similar dipole models, as well as similar cortical activity (measure-similarity). MPA fits the selected ICs into a 3D brain model comprised of a cubic space grid with 8-mm spacing according to normalized Montreal Neurological Institute (MNI) space. Cortical regions of interest were identified by the MPA toolbox by incorporating the probabilistic atlas of human cortical structures provided by the Laboratory of Neuroimaging project (Shattuck et al., 2008). Voxels that fell outside of the brain model (muscle artifacts, etc.) were excluded from the analysis. Note that the spatial resolution of EEG data compared to other brain-imaging methods such as fMRI and PET is relatively poor. It is important to practice caution when attributing brain activity to specific brain regions using EEG. The IC dipoles clustered via MPA have an associated probability of membership to a brain domain (Acar and Makeig, 2013).

We then calculated local convergence values, using an algorithm based on Bigdely-Shamlo et al. (2013) to deal with the multiple comparisons problem. Local convergence calculates the measure-similarity of dipoles within a given domain and compares them with randomized dipoles. In order to compare dipoles, a pairwise IC similarity matrix was created by estimating the signed mutual information between independent component-pair ERSP measure vectors, assuming a Gaussian distribution. As explained in detail by Bigdely-Shamlo et al. (2013), signed mutual information was estimated to improve the spatial smoothness of the obtained MPA significance value beyond determining similarity of dipoles through correlation. We used bootstrap statistics to obtain a significance threshold for convergence at each location of our 3D brain model. Following past literature, we set the raw voxel significance threshold to *p* < .001 (Bigdely-Shamlo et al., 2013; Chung et al., 2017).

Two relevant domains were analyzed: the right motor area, with the greatest concentration of dipoles consistent with right premotor and supplementary motor area (BA 6), and the left motor area, with the greatest concentration of dipoles consistent with left premotor and supplementary motor area (BA 6). For the right motor area, each participant contributed on average 2.27 (±1.27) ICs, with each participant contributing at least one IC, with a range from 1 to 5 ICs. For the left motor area, each participant contributed on average 2.18 (±1.17) ICs, with each participant contributing at least one IC, with a range of 1–4 ICs.

For each domain calculated by MPA, ERSPs were computed for each experimental condition. Within each domain, bootstrap statistics were used to assess differences in ERSP between conditions to uncover main effects of task and congruency. Differences at each power band were computed by projecting the ERSP for each condition to each voxel in the domain. For each subject, this projection was weighted by dipole density per voxel and then normalized by the total domain voxel density. Analysis of projected source measures were separated into discrete spatial domains by threshold-based Affinity Propagation clustering based on a similarity matrix of pair-wise correlations between ERSP measure values for each position. Following Chung et al. (2017), we used the maximal exemplar-pair similarity, which ranges from 0 to 10 to set a value of 0.8 (Bigdely-Shamlo et al., 2013; Chung et al., 2017; Ofori et al., 2015).

### 2.7 Data and code availability

The data and code for all analyses are available online at https://github.com/bentownsend11/Beta-band-power-is-an-index-of-multisensory-weighting-during-self-motion-perception.

## 3 Results

### 3.1 Behavioral results

We ran two 2 (input: visual-motion vs physical-motion) x 2 (direction: left heading vs right heading) ANOVAs to analyze participants’ accuracy and response time. Outliers were defined as trials with response times greater than three standard deviations above or below the mean in each condition, and were eliminated from all further analyses. Accuracy was high overall and there were no significant differences in accuracy between the physical-motion (*M* = 98.71, *SE* = 0.72) and visual-motion conditions (*M* = 99.88, *SE* = 0.08), *F*(1,9) = 2.56, *p* = .14, nor were there differences between right (*M* = 99.24, *SE* = 0.30) versus left heading directions (*M* = 99.40, *SE* = 0.44), *F*(1,9) = 0.19, *p* = .68.

The present study did uncover a significant difference in response times between the blocked physical-motion (*M* = 931 ms, *SE* = 91.20) and visual-motion conditions (*M* = 571 ms, *SE* = 43.68), *F*(1,9) = 23.42, *p* < .001, *η*_*p*_^2^ = 0.72. This difference was expected due to faster perceptual processing of visual inputs versus vestibular inputs (Barnett-Cowan and Harris, 2013). There were no significant differences in response time between right (*M* = 747 ms, *SE* = 62.16) versus left (*M* = 754 ms, *SE* = 60.61) headings, *F*(1,9) = 0.28, *p* = .61, and no input x direction interaction. These results are consistent with previous work reported in the literature but may be interpreted with caution due to the small sample size.

### 3.2 Oscillatory power

**Fig. 2:**
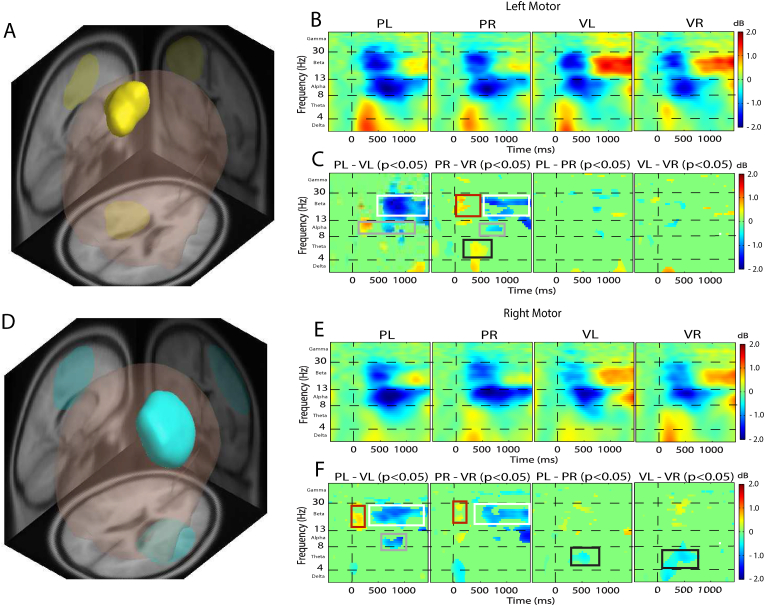
**(EEG Results).** Left motor area (Panels A, B, and C) and right motor area (Panels D, E, and F) identified by MPA and respective ERSP analysis. The ERSP plots show time (ms) across the x-axis and frequency of the EEG signal along the y-axis. Panels B (left motor) and E (right motor) show the associated ERSP plots for each condition. Panels C (left motor) and F (right motor) show the bootstrapped comparisons (p < .05) between each pair of conditions that are relevant to our hypotheses. ERS power is depicted in yellow/red, ERD power is depicted in blue, and green shows no difference in spectral power compared to baseline. **Left motor area: Panel A** shows a 3D representation of the brain with the yellow region representing the MPA domain with the greatest concentration of dipoles consistent with left premotor and supplementary motor area (BA 6). **Panel B:** ERSP plots for each condition. **Panel C:** Results of bootstrapped comparisons. PL-VL: the white square, showing significantly more beta ERS in the VL condition (note that due to subtraction PL-VL: Greater ERS power in VL is represented in blue). The grey square highlights a significant latency difference in alpha ERD between the PL and VL conditions. PR-VR: The red square highlights significantly more beta ERD early in the trial due to an earlier latency in the VR condition. The white square highlights more powerful beta ERS later in the trial for VR versus the PR condition. The grey square highlights significantly more alpha ERD in the PR condition. The black square highlights significantly more theta ERS in the PR condition. Contrasting PL-PR, and VL-VR show no robust differences in power. **Right motor area: Panel D** shows a 3D representation of the brain with the blue region representing the MPA domain with the greatest concentration of dipoles consistent with right premotor and supplementary motor area (BA 6). **Panel E:** ERSP plots for each condition. **Panel F:** Results of bootstrapped comparisons PL-VL: The red square highlights significantly more beta ERD early in the trial due to an earlier latency in the VL condition. The white square highlights more powerful beta ERS later in the trial for VL versus the PR condition. The grey square highlights significantly more alpha ERD in the PL condition. PR-VR: The red square highlights significantly more beta ERD early in the trial due to an earlier latency in the VR condition. The white square highlights more powerful beta ERS later in the trial for VR versus the PR condition. PL-PR: The black square highlights significantly more theta ERS for PR versus the PL condition. VL-VR: The black square highlights significantly more theta ERS for VR versus the VL condition. (For interpretation of the references to colour in this figure legend, the reader is referred to the Web version of this article.)

In Fig. 2 we show the left and right motor areas to provide side-by-side comparisons of how modality and direction of motion cues affected cortical activity. All ERSP is representative of a difference in oscillatory power compared to baseline (pre-trial) cortical activity, where an ERS (event-related synchronization) represents more spectral power than baseline and an ERD (event-related desynchronization) represents less spectral power than baseline. In Fig. 2, Panel A shows the left motor area, which has the highest dipole density in left premotor and supplementary motor areas (Brodmann area [BA] 6), and Panel D shows the right motor area, which has the highest dipole density in right premotor and supplementary motor areas (BA 6). In Panels B and E, we show the associated ERSP plots for each condition. Panels C and F show the bootstrapped comparisons between conditions within their respective motor areas.

#### 3.2.1 Power differences between modalities

*Theta-band activity:* Comparing physical vs visual rightward conditions, PR elicited greater theta ERS than VR (*p < .05*) from ∼400 ms to 550 ms post-stimulus in the left motor area (Panel C).

*Alpha-band activity:* Both left and right motor areas showed differences within the alpha-band in the visual-versus physical-motion conditions. In the left motor area, the VL condition, due to an earlier latency, elicited greater alpha ERD than PL (*p < .05*) from stimulus onset to ∼400 ms post stimulus. Whereas within a later time window, the PL condition elicited greater alpha ERD than VL (*p < .05*) from ∼600 ms to 1000 ms post-stimulus (Panel C). Comparing rightward conditions, PR elicited greater alpha ERD than VR (*p < .05*) from ∼600 ms to 900 ms post-stimulus onset (Panel C).

In the right motor area, the PL condition elicited greater alpha ERD than VL (*p < .05*) from ∼550 ms to ∼1000 ms post stimulus (Panel F), whereas no alpha band differences were observed between PR and VR in the right motor area (Panel F).

*Beta-band activity:* Each comparison resulted in significant differences in beta-band activity. In the left motor area, comparing leftward conditions, the VL condition elicited greater beta ERS than PL (*p < .05*) from ∼700 ms post stimulus onset to end of trial (Panel C). Comparing rightward conditions, VR elicited greater beta ERS than PR (*p < .05*) across a slightly longer but overlapping time window, from ∼500 ms to end of trial (Panel C). The VR condition elicited greater beta ERD compared to PR (*p < .05*) over an earlier time window from stimulus onset to ∼250 ms post stimulus onset.

In the right motor area, comparing leftward conditions, the VL condition elicited greater beta ERD than PL (*p < .05*) from stimulus onset to ∼250 ms post stimulus, and greater beta ERS than PL from ∼450 ms to end of trial (Panel F). Comparing rightward conditions, VR elicited greater beta ERD than PR (*p < .05*) from stimulus onset to ∼200 ms post stimulus, and greater beta ERS than PR from ∼550 ms to end of trial (Panel F).

#### 3.2.2 Power differences between headings

*Theta-band activity:* We found robust directional differences in the theta band only. The theta-band differences were observed in the right motor area, but not the left. In the right motor area the PR condition elicited more powerful theta ERS compared to PL (from ∼250 ms to 600 ms post-stimulus; Panel F). Similarly, VR elicited more powerful theta ERS compared to VL (from ∼100 ms to 750 ms post-stimulus; Panel F).

## 4 Discussion

As we move through the world, self-motion perception is a function of multisensory integration of visual, vestibular, proprioceptive, and auditory cues to self-motion. Observation of localized neural oscillations contributes to understanding of how ERSPs reflect the integration process. Multisensory integration works through a weighting process whereby more salient or reliable cues drive the perception of self-motion (Angelaki et al., 2009). These processes are most often studied by presenting simultaneous multisensory motion cues. For example, previous studies explored theta, alpha, and beta oscillations elicited in during a heading discrimination task in which participants attended to one or the other of simultaneous visual and physical motions cues (Townsend et al., 2019). Visual and physical cues were incongruent on half the trials, producing strong sensory conflict on the incongruent trials which increased the demand for multisensory weighting processes. A more powerful beta ERD was observed when attention was directed to the physical motion, whereas a more powerful beta ERS, and earlier induction of theta ERS and alpha ERD were observed when attention was directed to the visual motion. A limitation of the Townsend et al. (2019) study was that it did not include a condition in which visual (or physical) motion was presented alone, therefore it is not certain that the results observed in the attention conditions reflect a multisensory weighting process.

The present study aimed to address this gap in the literature by contrasting theta, alpha, and beta oscillations elicited in response to single modality visual-only versus physical-only motion cues. Thus, the multisensory weighting process was more heavily biased towards either visual or physical signals compared to the Townsend et al. (2019) study. An important motivation for the present study was to generate hypotheses about whether the theta, alpha and beta oscillations reflect a multisensory integration process or a more general process engaged during self-motion perception.

We observed modality differences in the theta and alpha bands localized to left and right motor areas. We suggest that theta ERS and alpha ERD may be associated with processes other than multisensory weighting because theta and alpha band power in the present study replicated Townsend et al. (2019) despite a large difference in demand for multisensory weighting between the two studies. In contrast, strong beta ERD and ERS responses support the hypothesis of Townsend et al. (2019) that beta oscillations are an index of processes that play a key role in multisensory weighting during visual-vestibular integration. We discuss beta oscillations, theta ERS, and alpha ERD in detail below.

### 4.1 Beta oscillations

The most notable modality-induced ERSP power and latency differences within the present study and Townsend et al. (2019) were in the beta band. The present study uncovered a significantly longer lasting and more powerful beta ERS (∼700 ms – end of trial), in the visual-motion condition compared to the physical-motion condition (∼850 ms – end of trial). The modality differences in the beta band observed by Townsend et al. (2019) were driven by a more powerful and longer-lasting beta ERD in the attend physical-motion conditions compared to attend visual-motion conditions. The ERSP power and time course differences in the beta band between the two studies could be due to the difference in multisensory weighting processes. Considering beta-band oscillations are so strongly linked with sensorimotor processing, it is intuitive that this difference in stimulus presentation and attention requirements (regardless of both experiments using the same stimuli) caused different ERSP power and time courses within the beta band.

Much research has shown that beta ERD is associated with motor output (Ofori et al., 2015; Woolrich et al., 2019), motor planning (Tzagarakis et al., 2010) and motor imagery (Pfurtscheller et al., 2005). Moreover, beta ERD amplitude, duration and onset time have been shown to be modulated by task parameters such as certainty of movement, or number of movement options (Tzagarakis et al., 2010). Beta ERD lasts until the movement or imagery is complete, and is typically observed bilaterally over sensorimotor areas (Salmelin and Hari, 1994; Stancak and Pfurtscheller, 1996). Beta ERS power rapidly increases if movement is not performed, for example after presentation of a No-Go signal (Alegre et al., 2004), or as soon as the motor output ceases (Baker et al., 1999). This increase in beta ERS power following the offset of movement is known as beta rebound, and it typically occurs 300–1000 ms post-movement (for review see Kilavik et al., 2013). Similar to the motor imagery beta ERD described by Pfurtscheller et al. (2005), the beta rebound has also been demonstrated in motor imagery tasks (Solis-Escalante, Mü;ller-Putz, Pfurtscheller, Neuper, 2012). One hypothesis is that the function of beta rebound is to recalibrate or reset the motor system to new conditions, in order to prepare for a subsequent movement (Gaetz and Cheyne, 2006). Once the beta rebound has expired, the beta oscillation cycle begins again with the preparation for a new movement. If the beta rebound were simply reflecting the recalibration of the motor system, we would not expect such a robust power difference between visual versus physical cues to motion, as we demonstrated in the present study. Alternatively, Townsend et al. (2019), suggested that the beta cycle might be engaged as part of a mechanism to suppress the processing of stimuli that are unattended or no longer require processing. For example, in the present experiment, the “no-motion” signal of the unstimulated sensory modality (i.e. the vestibular/proprioceptive systems during the visual-motion condition, or vice versa). The integration of the visual and vestibular systems is a subadditive process (Angelaki et al., 2009; Morgan et al., 2008). This robust beta rebound might reflect an inhibitory process during visual-vestibular integration in which the sensory information of the provided motion stimulus is weighted greater than the opposing no-motion signal. We believe the difference in beta rebound power between the two modalities may reflect visual bias in the visual-vestibular integration that has been reported by previous studies (e.g., Angelaki et al., 2009), considering the beta rebound was much stronger in the visual-only condition of the present study and the attend-visual condition of Townsend et al. (2019).

Finally, it should be noted that we did not find the longer lasting beta ERD in the physical condition compared to the visual condition that was found in Townsend et al. (2019). The 2019 study demonstrated a more powerful and longer-lasting beta ERD, starting at ∼600 ms and maintaining to the end of the trial in the attend-physical condition. We believe this different result could be due to the different attentional requirements of the task in Townsend et al. (2019) compared to the present study. The past study presented both visual- and physical-motion stimuli simultaneously and in conflict with one another on half of the trials, which required a greater need for sustained attention to the physical-motion stimulus during the attend-physical task. If attending to physical-motion stimuli elicits a beta ERD, then we would expect a longer-lasting beta ERD during sustained attention to the physical-motion stimulus, and that is exactly what was demonstrated in Townsend et al. (2019). In contrast, the present study only provided one sensory stimulus at a time. With minimal conflict between the visual and vestibular information, fewer attentional resources were required to complete the task, and intuitively we found shorter beta ERD at the beginning of the trials (∼150–700 ms). Combining our observations of beta ERS (rebound) and ERD, we believe that beta oscillations are critical to the weighting process during multisensory integration. The power of early onset beta ERD may reflect the attentional demands of unisensory or multisensory stimuli, while the power of the beta rebound may correlate with the magnitude of inhibition directed towards the lesser-weighted sensory modality during subadditive integration.

### 4.2 Theta ERS

Human (Kahana et al., 1999) and non-human (Shin, 2010; Welday et al., 2011) studies have shown that processes indexed by theta ERS are sensitive to spatial orientation and heading direction changes. These studies generally link theta ERS produced by grid cells and place cells distributed throughout the hippocampus and parahippocampal areas to the function of path integration (Burgess, 2008). A highly accepted hypothesis is that the entorhinal grid cells and hippocampal place cells work together to create an internal representation of the organism's location within its environment (Krupic, Bauza, Burton & O'Keefe, 2018). Place cells process external perceptual information to create an allocentric representation of the external environment, while grid cells process self-motion information, leading to a perception of one's dynamic egocentric position in relation to the external objects (O'Keefe and Burgess, 2005). According to the oscillatory interference model (Burgess, Barry & O'keefe, 2007), grid cell firing, and its modulation by self-motion, may result from two oscillations within the theta band which are distinguished by phase-change differences in response to the velocity of the moving organism. The phase of one theta oscillation becomes increasingly earlier as the animal moves through a given place field, while the other theta oscillation remains constant. Changes in the discrepancies of these theta oscillations' phase allows for the perception of changes in velocity and heading direction.

The current study found theta ERS to be sensitive to heading direction during both visual and physical self-motion perception. This effect, however, was only found in the right motor area. This lateralized effect is supported by past research, which has shown a right-lateralized brain network involved in spatial attention in humans (for review see Dieterich and Brandt, 2018). Studies focussed on spatial attention (for review see Bonato, 2012) and navigation (Buxbaum et al., 2008; Turton et al., 2009) in neglect patients shed light on the lateralized nature of spatial processing. Neglect patients typically fail to orient or respond to stimuli in the hemifield opposite of the acquired brain lesion (i.e., the contralesional side of space within a reference frame centered on the observer; Bonato, 2012). A study by Beis et al. (2004), found that 35–42% of stroke patients with a lesion to the right hemisphere suffered from neglect versus only 8–13% in patients with damage to the left hemisphere.

The present study also revealed a latency difference in theta ERS when comparing the time course of ERSP power between modalities, in which theta ERS was elicited later (100–550 ms) in the physical-only condition versus the visual-only condition (stimulus onset – 450 ms). This effect was similar to the effect found in Townsend et al. (2019). In both studies, theta ERS induction was earlier when visual motion information was the target stimulus (visual-motion only in the present study or attend-visual in the previous study). Considering the results of both of these experiments, it seems that this early theta ERS is not primarily driven by multisensory weighting processes, and is not specific to one sensory input (visual or vestibular). This finding also suggests that early theta ERS is likely not elicited by reflexive movements caused by physical motion. Based on previous literature (e.g., Peterson and Ferris, 2018), we would expect significant theta ERS power differences in the motor cortices if this were the case. Theta ERS seems to be engaged by sensory processing related to self-motion perception. In fact, this claim is supported by decades of research demonstrating that theta is an index of the initial stages of heading processing (for reviews see Başar et al., 2001 and Colgin, 2013).

Importantly, modality affected the time course of theta ERS similarly in both the present study and the Townsend et al. (2019) study, despite very different demands for multisensory weighting between the two studies. One conclusion is that the process indexed by theta ERS is occurring before visual-vestibular weighting takes place. Moreover, in the present study there was an interaction involving the right-lateralized heading differences observed for both the visual-only and physical-only modalities. The fact that both modality conditions showed similar right-lateralized theta-band changes points to this process being farther along the spatial processing timeline than merely sensing the stimulus. Taken together, this suggests that the early burst of theta ERS reflects a process that occurs after the sensation of the motion stimuli but is not affected by multisensory weighting. Although the limitations of spatial resolution with EEG recordings must be taken into account, the theta ERS oscillatory pattern could represent a contribution from activation of a network associated with place and grid cells from the parahippocampal area that are known to facilitate theta rhythms. Several studies have demonstrated that subsets of these cells are sensitive to visual-motion cues, physical-motion cues or a combination of both (Chen, King, Burgess & O'Keefe, 2013; Fattahi et al., 2018; Li et al., 2020).

### 4.3 Alpha ERD

The visual- and physical-motion conditions elicited alpha ERD with different latencies. Alpha ERD is currently understood to be associated with high focal cortical activation, while alpha ERS is associated with deactivation or inhibition, particularly within task-irrelevant brain areas (Klimesch, 2012). It has been demonstrated that engaging in perceptual judgment or increased attentiveness leads to an increase in alpha ERD power (Adrian and Matthews, 1934; Niedermeyer and da Silva, 2005). This association has been shown across a variety of cognitive tasks (and their respective brain areas), such as reading (Angelakis and Lubar, 2002), auditory oddball tasks (Yordanova et al., 2001), and observing the motor output of others (Avanzini et al., 2012). It is unsurprising then that alpha ERD is also found within the motor area during full-body self-motion perception in experiments such as the present study and Townsend et al. (2019). In a series of four large-scale EEG experiments, Fink et al. (2005), showed that alpha ERD power may simply reflect the attentional demands of the current task; the more demanding the task, the stronger the alpha ERD (i.e., stronger cortical activation). We found an earlier alpha ERD during the visual-motion task versus the physical-motion task. Research has shown that the perception of visual information is faster than vestibular and proprioceptive information (Barnett-Cowan and Harris, 2013). The differences in the timeline of alpha ERD power in the present study corresponds with the findings of Fink et al. (2005), and Barnett-Cowan and Harris (2013). If the visual-motion stimulus is perceived faster than the physical-motion stimulus, intuitively the attentional demands should be greater earlier in the trial during the visual-motion task compared to the physical-motion task, thus creating a latency difference in alpha ERD. In the present study, we believe this latency difference reflects the timing differences of when attentional resources are engaged during visual versus physical self-motion perception. Considering this alpha ERD is recorded from the motor area, it is possible that this process indexed by alpha ERD might be part of multisensory integration. However, based on the data from the present study and Townsend et al. (2019), it is clearly not affected by the difference in multisensory weighting demands across the two studies.

### 4.4 Limitations and future directions

We did not record electromyographic (EMG) signals to directly remove movement-related artifacts from any reflexive compensatory adjustments in response to the physical motion. It is likely that our EEG data were only minimally affected because in the time range in which these movements would occur our visual-only condition elicited similar cortical activity as the physical-motion condition, however we cannot exclude the possibility of movement artifacts. PEPs related to compensatory movements commonly show up in the first 100 ms after stimulus onset (Varghese et al., 2017). In that time window our spectral data showed only latency differences, with oscillations in the theta, alpha and beta band occurring earlier in the visual-only condition. Future studies should account for compensatory adjustments by recording EMG of the neck muscles. This is especially important if participants are moved along axes known to elicit strong compensatory adjustments (e.g., roll).

A second potential limitation involves the timing of the visual- and physical-motion stimuli. The duration of the visual motion stimulus was 700 ms whereas the duration of the acceleration phase of the physical motion stimulus was 330 ms followed by a 1330 ms washout. This is typical in a motion simulator environment due to technological and spatial limitations of the motion platform (Pinto et al., 2008). Typically, visual and physical motion are presented together; it works well because vestibular processes detect acceleration but not velocity. The acceleration of the washout is close to zero and does not contribute to the perception of acceleration in the heading or opposite direction (Fig. 1). Thus, the perception is acceleration in the heading direction followed by constant velocity associated with the optic flow. The present experiment decoupled the visual and physical stimuli, and there is a possibility that the washout may be more detectable perceptually, and thus contribute to the ERSP measures, than in a typical experiment that presents visual and physical simultaneously. Future experiments should address this systematically by comparing a set of acceleration-washout durations, within the range of technological and spatial limitations of the motion platform.

Finally, our heading discrimination task required participants to push a button in order to make a heading judgement. It is possible that the preparation and execution of thumb movements contributed to the cortical activity in the motor cortices. Response time differences in timing of motor output may be reflected in the observed oscillations. The heading judgement task satisfied important objectives to 1) ensure that participants attended to the motion cues to elicit the cortical activity, and 2) provide behavioural measures. Future designs might include a passive condition to examine whether motor responses affect the cortical activity between the visual and physical motion perception conditions.

## 5 Conclusion

The present study examined cortical activity elicited in response to single modality visual-only versus physical-only motion cues. An important motivation was to generate hypotheses about whether the theta, alpha and beta oscillations reflect a multisensory integration process or a more general process engaged during self-motion perception. Beta ERD and ERS responses support the hypothesis that beta oscillations index processes in multisensory weighting during visual-vestibular integration. Theta ERS and alpha ERD may be associated with processes other than multisensory weighting and are likely related to a more general cognitive process in self-motion perception.

## Declaration of competing interest

The authors declare no competing financial interests. Funding for this study was provided to JMS and MvM by The 10.13039/501100000038Natural Sciences and Engineering Research Council of Canada (RGPGP-2014-00051); and the 10.13039/501100000196Canada Foundation for Innovation (2009M00034). These funding sources had no involvement in the study design, the collection, analysis and interpretation of data, in the writing of the report, and in the decision to submit the article for publication.
