# Supplementary material for: Beta-band power is an index of multisensory weighting during self-motion perception
Source: Neuroimage Rep. 2022 May 28;2(3):100102. doi: 10.1016/j.ynirp.2022.100102 (PMC12172878; doi:10.1016/j.ynirp.2022.100102)
Supplement: Multimedia component 1 [file mmc1.docx]

**Supplementary Materials**

**
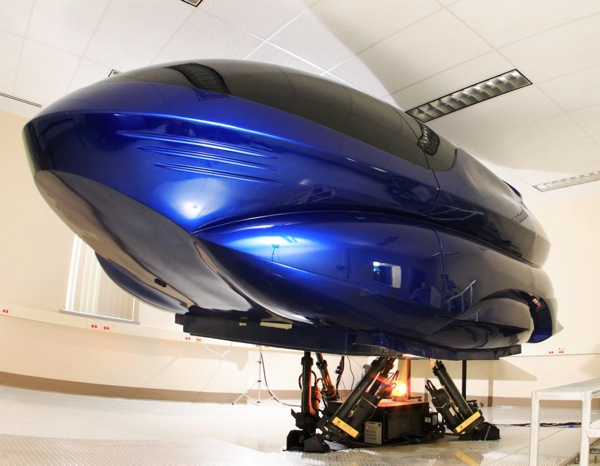
**

**Figure A1.** The motion simulator pod was supported by a MOOG © platform with six-degrees-of-freedom motion (MOOG series 6DOF2000E).


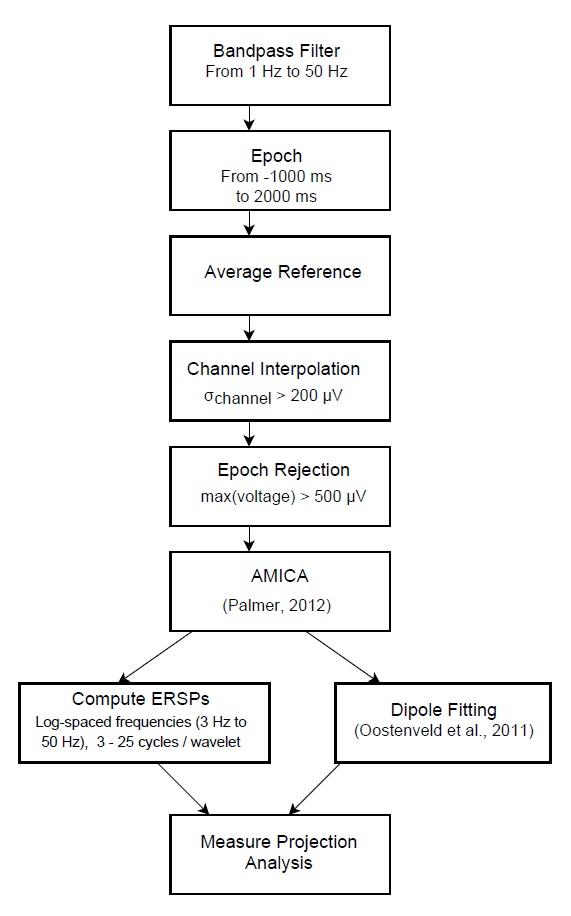


**Figure A2.** This flowchart illustrates the signal processing pipeline. The Measure Projection Analysis (MPA) pipeline is described in section 2.9 (ERSP Measure Projection Analysis), and a flowchart can be found in Bigdely-Shamlo, Mullen, Kreutz-Delgado & Makeig, 2013.
